# Dendritic cells loaded with exosomes derived from cancer stem cell‐enriched spheroids as a potential immunotherapeutic option

**DOI:** 10.1111/jcmm.16401

**Published:** 2021-02-25

**Authors:** Marzieh Naseri, Margot Zöller, Jamshid Hadjati, Roya Ghods, Ehsan Ranaei Pirmardan, Jafar Kiani, Leila Eini, Mahmood Bozorgmehr, Zahra Madjd

**Affiliations:** ^1^ Oncopathology Research Center Iran University of Medical Sciences (IUMS) Tehran Iran; ^2^ Department of Molecular Medicine Faculty of Advanced Technologies in Medicine Iran University of Medical Sciences (IUMS) Tehran Iran; ^3^ Section Pancreas Research University Hospital of Surgery Heidelberg Germany; ^4^ Department of Immunology School of Medicine Tehran University of Medical Sciences (TUMS) Tehran Iran; ^5^ Department of Radiology Molecular Biomarkers Nano‐imaging Laboratory Brigham & Women's Hospital Harvard Medical School Boston MA USA; ^6^ Department of Basic Science Faculty of Veterinary Science and Research Branch of Islamic Azad University Tehran Iran

**Keywords:** anti‐tumour response, colorectal cancer (CRC), dendritic cells (DCs), exosomes derived from cancer stem cell‐enriched spheroids (CSC_enr_‐EXOs), immunotherapy

## Abstract

Cancer stem cells (CSCs) are responsible for therapeutic resistance and recurrence in colorectal cancer. Despite advances in immunotherapy, the inability to specifically eradicate CSCs has led to treatment failure. Hence, identification of appropriate antigen sources is a major challenge in designing dendritic cell (DC)‐based therapeutic strategies against CSCs. Here, in an in vitro model using the HT‐29 colon cancer cell line, we explored the efficacy of DCs loaded with exosomes derived from CSC‐enriched colonospheres (CSC_enr_‐EXOs) as an antigen source in activating CSC‐specific T‐cell responses. HT‐29 lysate, HT‐29‐EXOs and CSC_enr_ lysate were independently assessed as separate antigen sources. Having confirmed CSCs enrichment in spheroids, CSC_enr_‐EXOs were purified and characterized, and their impact on DC maturation was investigated. Finally, the impact of the antigen‐pulsed DCs on the proliferation rate and also spheroid destructive capacity of autologous T cells was assessed. CSC_enr_‐EXOs similar to other antigen groups had no suppressive/negative impacts on phenotypic maturation of DCs as judged by the expression level of costimulatory molecules. Notably, similar to CSC_enr_ lysate, CSC_enr_‐EXOs significantly increased the IL‐12/IL‐10 ratio in supernatants of mature DCs. CSC_enr_‐EXO‐loaded DCs effectively promoted T‐cell proliferation. Importantly, T cells stimulated with CSC_enr_‐EXOs disrupted spheroids' structure. Thus, CSC_enr_‐EXOs present a novel and promising antigen source that in combination with conventional tumour bulk‐derived antigens should be further explored in pre‐clinical immunotherapeutic settings for the efficacy in hampering recurrence and metastatic spread.

## 1 INTRODUCTION

Colorectal cancer (CRC) is one of the most frequent malignancies and the leading cause of cancer death worldwide.^1^, ^2^ Despite efficient clinical interventions in early stages of CRC including surgery, chemotherapy and radiotherapy, at later stages they frequently are palliative and improve patients' life quality, but survival‐influencing tumour recurrence and metastasis still account for a high mortality rate.^3^, ^4^ Immune responses are well known as the vanguard of inherent anti‐cancer strategies, and progress in immunotherapeutic approaches has improved the efficacy of cancer treatment over the past decades.^5^, ^6^ However, the overall outcome is limited and unsatisfactory due to the inability of treatment strategies to target cancer stem cells (CSCs). Accumulating evidences suggest that tumour mass‐resident CSCs, a rare population of heterogeneous tumour cells, which display tumour initiating and self‐renewal capacity, and account for drug resistance, impose treatment failure.^7^, ^8^, ^9^ This issue precludes curative cancer treatment through tumour recurrence following therapy. Therefore new therapeutic strategies selectively targeting this particular stem‐like population are warranted.

A variety of immunological modalities have been utilized for CSCs eradication; these include targeting of CSC‐specific antigens (Ags) and niche, adoptive CSC‐primed T‐cell therapy, stimulation of innate immune responses and CSC lysate vaccines.^10^, ^11^, ^12^, ^13^, ^14^ In this respect, dendritic cells (DCs) as specialized antigen‐presenting cells (APCs) and effective initiators of adaptive immune responses, have played a pivotal role in priming and boosting anti‐tumour immune responses and developing cancer vaccines.^15^, ^16^ Emerging studies have demonstrated that CSC‐based DC (CSC‐DC) vaccines can target CSCs by promoting the induction of cytotoxic T cells (CTLs), leading to inhibition of tumour growth and relapse rate reduction. In addition, these vaccination modalities harnessed lung metastases, reduced tumour size and prolonged survival rates in animal tumour models through induction of interferon (IFN)‐γ production and activation of humoural and cellular immune responses against CSCs with no major adverse effects such as autoimmune reactivity.^17^, ^18^, ^19^, ^20^, ^21^, ^22^, ^23^ DC targeting of CSCs has been demonstrated to be advantageous overutilization of DC vaccines pulsed with either tumour bulk or parent cell lysate.^19^, ^20^, ^21^ These findings suggest the potential capacity of the immune system and in particular of DC‐based vaccines in eradicating CSCs.

Due to the limited and inadequate response rates induced by CSC lysates, identification of proper antigen sources for CSCs targeting is warranted. One option to improve cancer immunotherapy may rely on DC loading with tumour‐derived exosomes (TEXs). TEX, membrane nanovesicles (30‐140 nm), are released by tumour cells and were described to prepare the tumour microenvironment and pre‐metastatic niche in favour of tumour progression, metastasis and immune escape.^24^, ^25^, ^26^, ^27^, ^28^ In spite of their role in immune suppression, TEXs are enriched in both tumour antigens and costimulatory molecules and can induce anti‐cancer immunity,^29^, ^30^, ^31^ particularly when presented by DC. TEXs are taken up by DCs, induce their maturation, and capacity to stimulate antigen‐specific CTLs and IFN‐γ delivery.^32^, ^33^, ^34^ It is important to note that uptaken antigens are digested and exclusively loaded into newly generated MHCII molecules including the phenomenon of cross‐priming. Accordingly, DC‐TEXs vaccination inhibited tumour growth and improved the survival rate in several protective and therapeutic tumour vaccination models.^32^, ^35^, ^36^, ^37^ TEXs‐loaded DCs have been shown to elicit superior anti‐tumour immune responses compared to cell lysate‐loaded DC in vivo and in vitro.^32^, ^38^, ^39^, ^40^, ^41^, ^42^ However, the efficacy of DC‐based therapy using CRC‐derived TEXs vs CSCs‐derived exosomes (CSC‐EXOs) remains to be determined.

As the first step towards developing an effective CSC‐DC vaccine in CRC, we searched for an optimal antigen source in an in vitro model. TEXs are enriched in tumour antigens. Thus, we speculated that exosomes derived from CSC‐enriched populations may also be enriched in CSC‐selective antigens and thereby become particular therapeutic targets and most valuable immunogenic sources for DC loading. To our knowledge, thus far, there is no published report investigating the immunogenic potency of CSC_enr_‐EXOs in the context of anti‐tumour responses; here, we explored whether DCs loaded with exosomes derived from CSC_enr_‐EXOs could promote in vitro stimulation of T lymphocytes against colorectal cancer stem cells. DCs were also loaded with CSC‐enriched spheroid lysate, HT‐29 lysate and HT‐29‐EXOs as other potential Ag sources. Our preliminary results approved that CSC‐enriched spheroid‐derived EXOs do not interfere with phenotypic and functional maturation of DCs. Importantly, DCs loaded with CSC_enr_‐EXOs as a novel Ag source stimulated T‐cell proliferation and CSC‐directed cytotoxicity.

## 2 MATERIALS AND METHODS

### 2.1 Cell line and CSC‐enriched spheroid culture

HT‐29 colon adenocarcinoma cell line was obtained from the Iranian Biological Research Center (IBRC). Cells were maintained in high glucose Dulbecco's modified Eagle's medium (DMEM, Gibco, Germany) supplemented with 10% foetal bovine serum (FBS, Gibco, Germany), 1% non‐essential amino acids (Gibco, Germany), 2 mmol/L l‐glutamine (Gibco, Germany) and 100 U/mL penicillin, 100 µg/mL streptomycin (Biowest, France) at 37°C in 5% CO_2_ in a humidified incubator. For spheroids culture, 70%‐80% confluent HT‐29 cells were detached with 0.05% trypsin/EDTA (Gibco, Germany) and washed twice with PBS and serum‐free media. The single cells were re‐suspended (5 × 10^3^ or 10 × 10^3^) in 25 μL drops of serum‐free DMEM/F12 medium (Gibco, Germany) supplemented with 1% non‐essential amino acids, 2 mmol/L l‐glutamine, 2% B27 supplement (Gibco, Germany), 20 ng/mL epidermal growth factor (EGF, PeproTech, USA) and 10 ng/mL of basic fibroblast growth factor (bFGF, PeproTech, USA). Drops were dispensed on the lid of petri dishes containing 5 mL PBS to prevent dehydration. The lids were carefully inverted and hanging drop cultures were maintained at 37°C, 5% CO_2_ and 95% humidified incubator for 96 hours. Drops containing spheroids were harvested by washing with gentle shaking of media and transferred onto 1.2% sterile poly‐HEMA (Sigma, USA) coated dishes for six additional days. Half of the culture medium was exchanged with fresh media supplemented with 2% B27, EGF and bFGF every other day.

### 2.2 RNA isolation and quantitative PCR

To analyse the expression of *KLF4*, *SOX2*, *NANOG* and *OCT4* key stemness genes, the HT‐29 parental and spheroid cells were washed thrice with cold PBS and total RNAs were isolated using RNeasy Mini Kit (Qiagen, Germany) according to the manufacturer's instruction. To remove genomic DNA contamination, RNA samples were treated with DNase I. RNA quantity and integrity was determined by Nanodrop (ThermoFisher Scientific, USA) and an agarose gel. cDNA was generated using cDNA synthesis kit (GeneAll, Korea). Real‐time polymerase chain reaction (RT‐qPCR) was performed with the SYBR Premix Ex Taq II real‐time PCR kit (TaKaRa, Japan) on the Rotor‐Gene Q LightCycler (Qiagene, Germany). The house‐keeping gene encoding glyceraldehyde‐3‐phosphate dehydrogenase (*GAPDH*) was used as the internal reference gene. Primers are listed in Table 1.

**TABLE 1:** Primers used for real‐time PCR

Gene groups	Gene name	Primer Sequence (5′→3′)
Housekeeping gene	GAPDH	F‐CATGAGAAGTATGACAACAGCCT R‐AGTCCTTCCACGATACCAAAGT
Stemness genes	NANOG	F‐AGCTACAAACAGGTGAAGAC R‐GGTGGTAGGAAGAGTAAAGG
	SOX2	F‐AATGGGAGGGGTGCAAAAGAGG R‐GTGAGTGTGGATGGGATTGGTG
	KLF4	F‐CCTCGCCTTACACATGAAGAG R‐CATCGGGAAGACAGTGTGAAA
	OCT4‐A	F‐GTGGAGAGCAACTCCGATG R‐TGCAGAGCTTTGATGTCCTG

### 2.3 Exosome purification and cell lysate preparation

HT‐29 cells were grown up to 70% confluence in complete medium (DMEM/High glucose + 10% FBS). The medium was discarded and the cells were washed three times with PBS and cultured in DMEM supplemented with 10% Gibco™ exosome‐depleted FBS for 48 hours, when the culture supernatant was collected. Due to the serum‐free culture condition of spheroids, the conditioned medium was harvested after 10 days of culture. After centrifugation (300 *g* for 10 minutes) to remove cellular debris, the conditioned mediums were concentrated by a 100‐kD molecular weight cut‐off (MWCO) Amicon ultra capsule filter (Millipore, USA). Exosome purification was performed through precipitation by size exclusion chromatography using the Exo‐spin™ kit (EXO1‐8, Cell Guidance Systems, UK) according to the manufacturer's instruction. Purified exosomes were pooled. For cell lysate preparation, HT‐29 and CSC‐enriched spheroid cells were harvested and washed three times with PBS. The cell lysates were obtained by ten freeze‐thaw cycles using liquid nitrogen and a 37°C water bath and then were centrifuged at 20 000 *g* for 20 minutes to remove the cellular debris. The total protein concentration of isolated exosomes and cell lysates was determined by BCA protein assay kit (Takara, Japan) and stored at −80°C until use.

### 2.4 Exosome characterization

#### 2.4.1 Dynamic light scattering (DLS)

Exosomes were diluted in PBS and the diameter of the purified exosomes was determined by dynamic light‐scattering measurements (Malvern, UK). Exosomes derived from HT‐29 tumour cells and CSC‐enriched spheroids were termed as HT‐29‐EXO and CSC_enr_‐EXO, respectively.

#### 2.4.2 Scanning electron microscopy (SEM)

To observe exosomes morphology, purified exosomes were fixed with 2.5% glutaraldehyde in PBS for 30 minutes and then were dehydrated using a gradient of ethanol (50%, 70%, 80%, 90% and 100%). To prevent rapid evaporation of the fixative, slides were put on top of petri dishes filled with PBS. Finally, samples were coated with gold‐palladium and observed by a scanning electron microscope (SEM, Seron Technology, AIS‐2100, Korea).

#### 2.4.3 Western blotting

To characterize the exosomes, purified exosomes and cell extracts were lysed in the RIPA lysis buffer (150 mmol/L NaCl, 1% Triton X‐100, 0.5% sodium deoxycholate, 1% SDS, 50 mmol/L Tris (pH = 8)) and a protease inhibitor cocktail (Sigma, USA). After lysis, 15 µg of each sample was re‐suspended in reducing sodium dodecyl sulphate (SDS) loading buffer and incubated for 5 minutes at 95°C. Thereafter the samples were subjected to 12% SDS‐polyacrylamide gel electrophoresis. Following electrotransfer of separated proteins onto a polyvinylidene fluoride (PVDF) transfer membrane and a blocking step, the transferred proteins were immunodetected using the rabbit anti‐human anti‐CD63 and anti‐CD81 primary antibody (Exosomes Antibodies Array & ELISA Kits (EXOAB‐KIT‐1, System Biosciences (SBI), UK, 1:1000) overnight, followed by incubation with appropriate goat anti‐rabbit IgG horseradish peroxidase (HRP)‐coupled secondary antibody (1:20 000) for 1 hour at room temperature. The blot was subsequently developed using a chemiluminescent HRP substrate and chemiluminescence was detected using a LAS3000 instrument (Fujifilm, Japan). The primary antibody was omitted in the control group and rabbit IgG was used as isotype control.

### 2.5 Generation of monocyte‐derived DCs

Human peripheral blood mononuclear cells (PBMCs) were separated using Ficoll‐Paque (Inno‐trian, USA) density gradient centrifugation at 400 *g* for 20 minutes from leukapheresis products (buffy coats) of healthy donors (provided by Iran Blood Transfusion Organization (IBTO), Tehran, Iran). The CD14^+^ monocyte‐enriched fractions were purified using the negative selection antibody‐coated magnetic beads (human monocyte isolation kit II (Miltenyi Biotec, USA). For DC generation, the CD14^+^ cells (1 × 10^6^ cells/mL) were cultured in RPMI‐1640 medium (Invitrogen, USA) supplemented with 10% FBS, 2 mmol/L l‐glutamine, 100 U/mL penicillin, 100 µg/mL streptomycin and 1% non‐essential amino acid in the presence of 50 ng/mL recombinant human granulocyte macrophage colony‐stimulating factor (GM‐CSF, PeproTech, USA) and 20 ng/mL recombinant human interleukin (IL)‐4 (PeproTech, USA). Every 3 days, half of the medium was refreshed with double concentration of GM‐CSF and IL‐4. At day six of culture, 50 µg/mL of each antigen group including CSC‐EXOs, CSC lysate, HT‐29‐EXOs and HT‐29 lysate were added to each DC well (Table 2). After 4 hours, 50 ng/mL lipopolysaccharide (LPS, Sigma, USA) was added to induce complete DC maturation. In some wells immature DCs were only treated with LPS as the positive control group (LPS‐alone), and also in some wells immature DCs were treated with PBS instead of Ag or LPS (negative control). After a 48 hour‐incubation at 37°C, mature exosome‐ or lysate‐loaded DCs were harvested (day 8) and used as APCs.

**TABLE 2:** Antigen source groups derived from HT‐29 colon cancer cell line and used to pulse DCs

Antigen source groups*	Antigen type
HT‐29 Lysate	Lysate prepared from HT‐29 colon cancer cell line
HT‐29‐EXOs	Exosomes obtained from HT‐29 colon cancer cell line
CSC Lysate	Cancer stem cells enriched using spheroid from HT‐29 colon cancer cell line
CSC‐EXOs	Exosomes obtained from CSCs of HT‐29 colon cancer cell line

* Monocyte‐derived DCs were pulsed with all antigen groups, separately. LPS was added along with the antigens in all groups.

### 2.6 Phenotypic characterization of CSCs and DCs

DCs were stained with a panel of antibodies for expression of cellular surface molecules and then analysed by flow cytometry. The following fluorochrome‐labelled antibodies were used: PE anti‐human CD40 and CD83, APC anti‐human CD86 and HLA‐DR, Alexa Fluor 488 anti‐human CD14 and PerCP/Cy5.5 anti‐human CD1a (all from Biolegend, USA) for 30 minutes at 4°C. Anti‐mouse Ig, κ/Negative control compensation particles set (BD, USA) was used to optimize fluorescence compensation settings.

To evaluate the expression of putative CSC markers on spheroid cells, anti‐CD44 (abcam, USA), anti‐CD133 (abcam, USA), anti‐CD166 (abcam, USA) and anti‐DCAMKL1 (abcam, USA) primary antibodies and FITC‐goat anti‐rabbit IgG (Santa Cruz biotechnology, USA) secondary antibody were used. The tubes were run on an Atuune NxT flow cytometer (Thermo Fisher Scientific, USA) and analysed using FlowJo VX software.

### 2.7 Cytokine release from DCs

After the 8‐day cultures for generation of mature DCs, supernatants from all groups were collected and analysed for cytokine concentration. The level of IL‐12p40 and IL‐10 in the supernatants was determined by enzyme‐linked immunosorbent assay (ELISA, R&D system, USA) according to the manufacturer's protocol.

### 2.8 T‐cell proliferation assay

CD14^‐^ cells obtained from flushing of LS columns (Miltenyi Biotec, USA) after immunomagnetic monocyte isolation were washed twice with PBS and cryopreserved in FBS with 10% dimethylsulphoxide (DMSO, Sigma, USA) at −80°C for 1 day and then stored in liquid nitrogen until use. Thawed CD14^‐^ cells were cultured for 2 hours and the non‐adherent portion was considered as T cells. T cells were stimulated with autologous DCs at ratio of 10:1 (T:DC) in the presence of 10 ng/mL IL‐2 (PeproTech, USA). Half of the media was replaced every 3 days with fresh media containing 20 ng/mL IL‐2. On day eight, stimulated T cells were stained with carboxyfluorescein diacetate succinimidyl ester (CFSE, CFSE cell division tracker kit, BioLegend, USA) according to the manufacturer's instruction. CFSE‐labelled T cells (5 × 10^5^ cells) were restimulated under the same condition using 5 × 10^4^ DCs (10:1 T:DC) for 5 days. After the 5‐day co‐culture (second stimulation), T cells were washed in cold PBS. The proliferation rate was assessed using flow cytometry. In this regard, using Flowjo software (version. 7) the division index was calculated as the average number of cell divisions that a cell in the original population had undergone (the average also takes into account the undivided cells).

### 2.9 Measurement of spheroid destruction in co‐culture with activated T cells

Co‐culturing of spheroids with activated T cells was performed as previously described.^43^ Briefly, T cells were stimulated twice on days zero and eight of co‐culture using DCs at the ratio of 10:1 (T:DC); Thereafter, the functional capacity of activated T cells was investigated via running co‐cultures between T cells and HT‐29‐derived spheroids for 24 hours. The rate of destruction was examined via measuring the diameter of spheroids using Image J software (IJ 1.46r version, NIH, USA) on the phase‐contrast microscope pictures. More than 20 independent microscopic fields from spheroids of each group were counted.

### 2.10 Statistical analysis

All experiments were performed at least three times and data are reported as mean values ± SD. To determine the statistical significance of the real‐time PCR and flow cytometery experiments used to confirm CSC enrichment, paired Student's t *test* was used. The comparison of differences between groups in the case of other experiments was performed with one‐way ANOVA. All statistical analyses were carried out using GraphPad Prism version 8.0 for Windows (GraphPad Software, La Jolla, CA, USA, www.graphpad.com). *P* values less .05 were considered as statistically signiflcant and values less than .01, .001, .0001 were shown using **, *** and ****, respectively, on the graphs.

## 3 RESULTS

### 3.1 Preparation of CSC‐enriched spheroids

**FIGURE 1:**
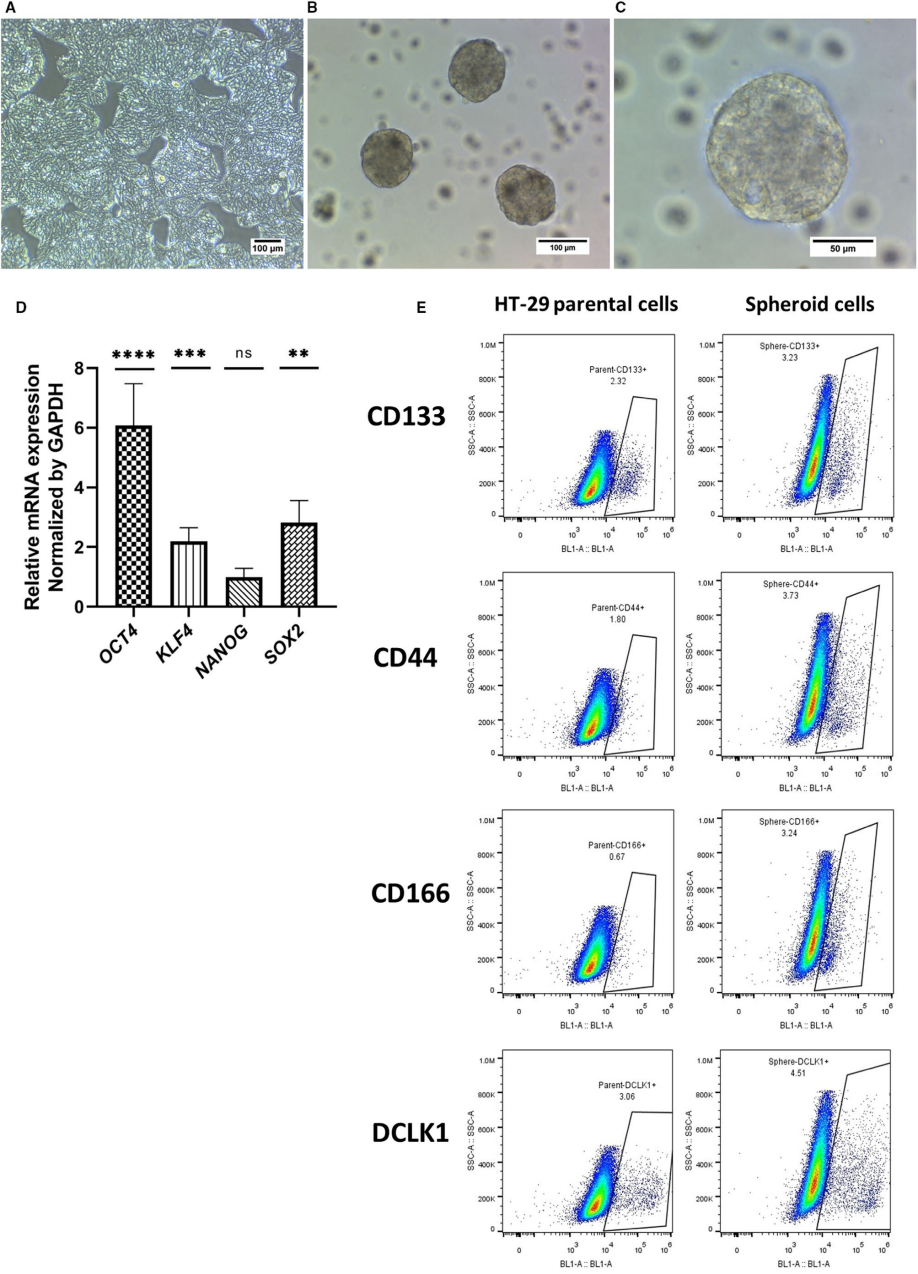
CSC enrichment within spheroids. (A) Representative images showing the morphological features of parental HT‐29 cell line and (B and C) their derived spheroids. (D) Real‐time PCR analysis showed significantly elevated relative expression levels of *OCT4, KLF4* and *SOX2* stemness genes in spheroids compared to parental cells normalized to *GAPDH* and, (E) representative flow cytometry plots confirmed the increased expression level of the putative colon CSC markers in spheroids compared to their differentiated counterparts. Data from real‐time PCR are presented as mean ± SD (n = 6)

Colon cancer spheroids were generated from the HT‐29 cell line in serum‐free and non‐adherent condition (Figure 1A‐C). Enrichment of cancer stem‐like cells was confirmed by investigating the expression of *SOX2*, *KLF4*, *NANOG* and *OCT4* key stemness genes using real‐time PCR. Spheroids showed a significant increase in *OCT4, KLF4* and *SOX2* gene expression compared to parental HT‐29 cells (Figure 1D). To further verify CSC enrichment, the CRC‐CSC surface markers CD44, CD133, DCLK1 and CD166 were assessed by flow cytometry. As shown in Figure 1E and Table 3, single cell suspension from spheroids revealed all 4 markers being expressed at a higher percentage, but only the increase in CD133^+^ and CD166^+^ cells was significantly higher than in HT‐29 cells.

**TABLE 3:** The expression percentage of CD44, CD166, CD133 and DCLK1 common CRC‐CSCs markers in spheroids compared to their HT‐29 parental cells

Sample cells	CD44%	*CD133%	DCLK1%	*CD166%
HT‐29 parental cells	2.50 ± 0.75	2.05 ± 0.45	2.46 ± 0.98	1.53 ± 1.03
Spheroid cells	6.13 ± 2.98	3.26 ± 0.03	4.47 ± 0.15	5.27 ± 1.97

Note

The data were reported as mean ± SD% of three independent flow cytometry experiments. There was a significant increase in the expression of CD133 and CD166 CSCs markers in spheroids when compared to parental cells, (*P < .05).

### 3.2 Characterization of exosomes derived from HT‐29 and CSC‐enriched spheroids

**FIGURE 2:**
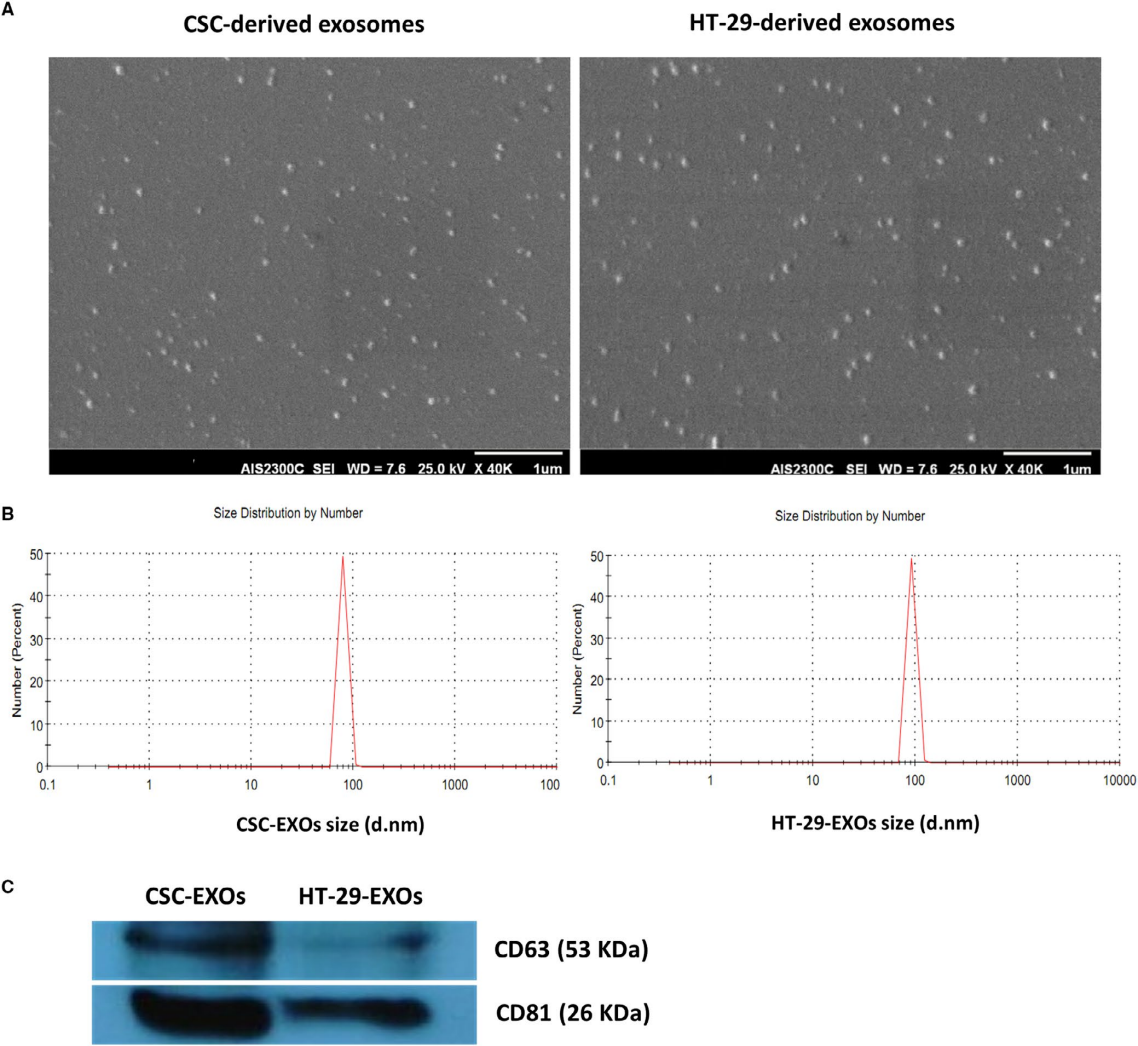
Characterization of exosomes purified from culture supernatant of HT‐29 and spheroid cells. (A) Scanning electron micrographs of the exosomes from HT29 parental cells and spheroids derived thereof (scale bar = 1 µm). (B) Size distribution of HT‐29‐ and CSC‐derived exosomes, measured by dynamic light scattering, showed diameter ranges of 79.55 ± 8.47 and of 92.17 ± 9.85 nm for CSC‐ and HT‐29‐EXOs, respectively. (C) Western blot analysis of the exosome markers CD63 and CD81 in CSC‐ and HT‐29‐EXOs

After confirming CSCs enrichment in spheroids, exosomes were purified from the culture supernatant of both HT‐29 and spheroid cells. Both exosome groups, as observed by SEM, displayed relatively uniform size and typical round morphology (Figure 2A) and consisted of a population of homogeneous single vesicles with Z‐average of 100.18 and 133.84, and size range of 79.55 ± 8.47 and of 92.17 ± 9.85 nm diameter as determined by dynamic light scattering (DLS) for CSC_enr_‐ and HT‐29‐EXOs, respectively (Figure 2B). The expression of CD63 and CD81 exosomal protein markers in isolated exosomes was detected by Western blot analysis. Surprisingly, the expression of both tetraspanin markers in CSC_enr_‐EXOs exceeded that in HT‐29‐EXOs (Figure 2C). Nonetheless, these results confirmed the nature and purity of the isolated exosomes.

### 3.3 DC maturation status in the presence of exosomes and cell lysates of HT‐29 cells and CSC‐enriched spheroids

**FIGURE 3:**
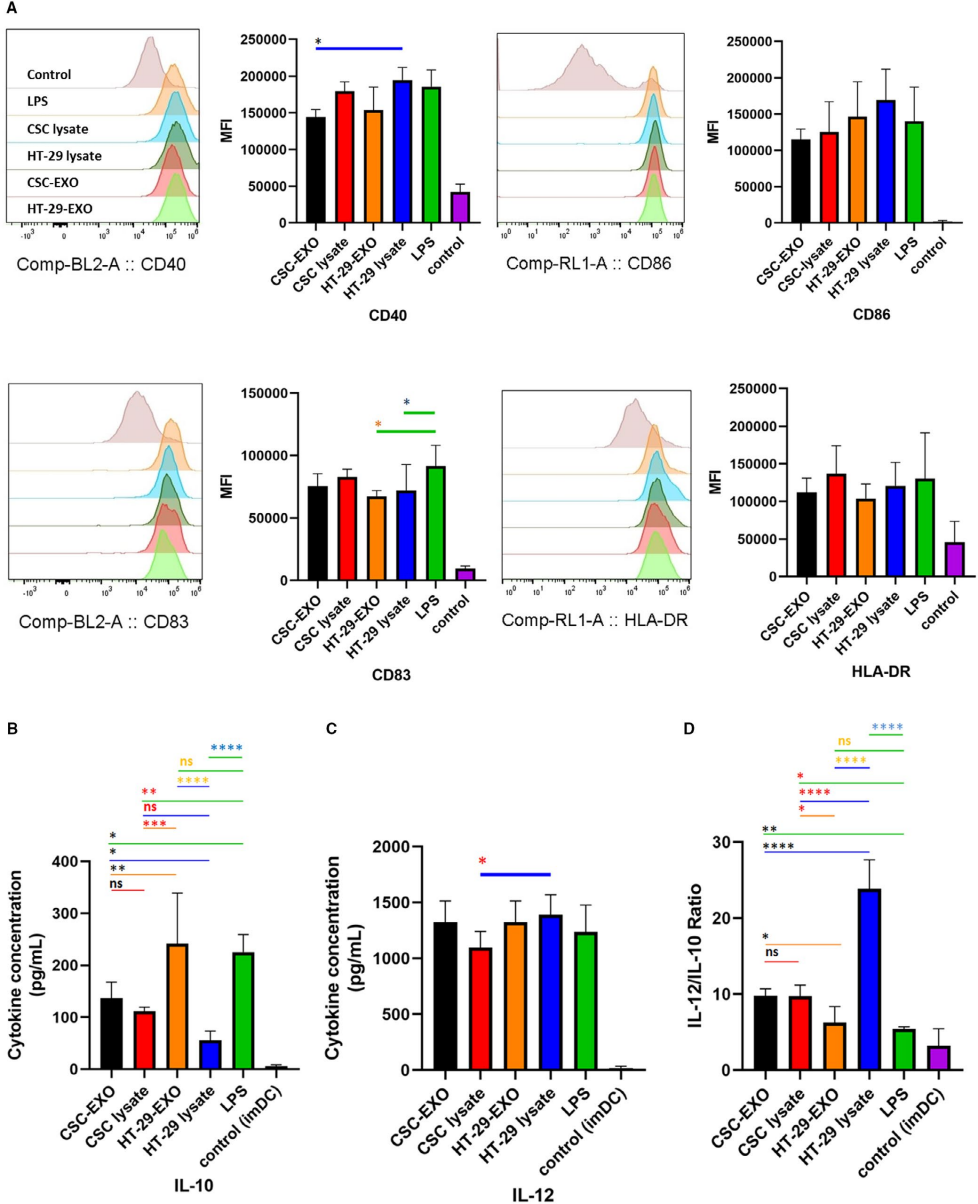
The impact of different antigen sources on DCs maturation. Immature DCs were induced for maturation in presence of CSC_enr_‐EXOs and other antigen sources separately; the expression of surface DC activation markers was investigated using flow cytometery. (A) The bar diagrams indicate mean MFI ± SD of DC markers (n = 4); histograms show the expression of each markers in one representative experiment. All DC groups including CSC_enr_‐EXOs showed significant differences in mature DC marker expression compared to control group (imDC). In addition, (B) IL‐10 and (C) IL‐12 cytokine secretion, (D) and the IL‐12/IL‐10 ratio in the supernatants of cultured DCs were tested using ELISA. The data represent mean ± SD from four independent experiments

Loading of immature DCs with tumour lysates or EXOs promotes DC maturation.^31^, ^44^ To investigate the potential impact of CSC_enr_‐EXOs on the maturation status of DCs, immature DCs were loaded with CSC_enr_‐EXOs and in separate groups with CSC_enr_ lysate, HT‐29 lysate and HT‐29‐EXOs (Table 2) as Ag sources in the presence of LPS. DCs loaded with LPS alone were considered as the positive control (LPS‐alone). Morphological monitoring indicated branched projections on DC from all tested groups (data not shown). These supposedly mature DCs were analysed for the expression of CD40, CD83, CD86 and HLA‐DR, as well as the concentration of IL‐12 and IL‐10 in cultured supernatants. The expression level of CD86, HLA‐DR and CD40 was significantly increased in antigen‐stimulated DCs with no or only borderline significant difference between the four Ag‐pulsed (Table 2) and the LPS‐alone groups (Figure 3A). However, the HT‐29‐pulsed DCs showed a significantly higher expression of CD40 compared to the CSC_enr_‐EXO group. As related to CD83, both HT‐29 lysate‐ and HT‐29‐EXO groups showed a significantly lower expression compared to the typical maturation group (LPS‐alone), whereas the CSC_enr_ lysate‐ and CSC_enr_‐EXO‐pulsed DCs did not significantly differ from the LPS‐alone group (Figure 3A).

A different picture emerged evaluating IL‐12 and IL‐10 secretion. All antigen groups supported IL‐12 secretion with no significant difference to the LPS group, but HT‐29 lysates being slightly more efficient than CSC_enr_ lysates. Instead, with the exception of HT‐29‐EXOs, IL‐10 secretion was suppressed compared to the LPS group (Figure 3B,C). Accordingly, the IL‐12 to IL‐10 ratios of CSC_enr_‐EXO‐, CSC_enr_ lysate‐ and HT‐29 lysate‐loaded DCs significantly exceeded that of the LPS group, the ratio being highest for HT‐29 lysate‐loaded DCs. With the exception of HT‐29 lysates, differences in the IL‐12 to IL‐10 ratios between the three remaining antigen‐pulsed DCs were not or only borderline significant (Figure 3D). In conclusion, there is no evidence for CSC_enr_‐EXO exerting a negative impact on DC maturation.

### 3.4 CSC_enr_‐EXOs‐pulsed DCs induce autologous T‐cell proliferation

**FIGURE 4:**
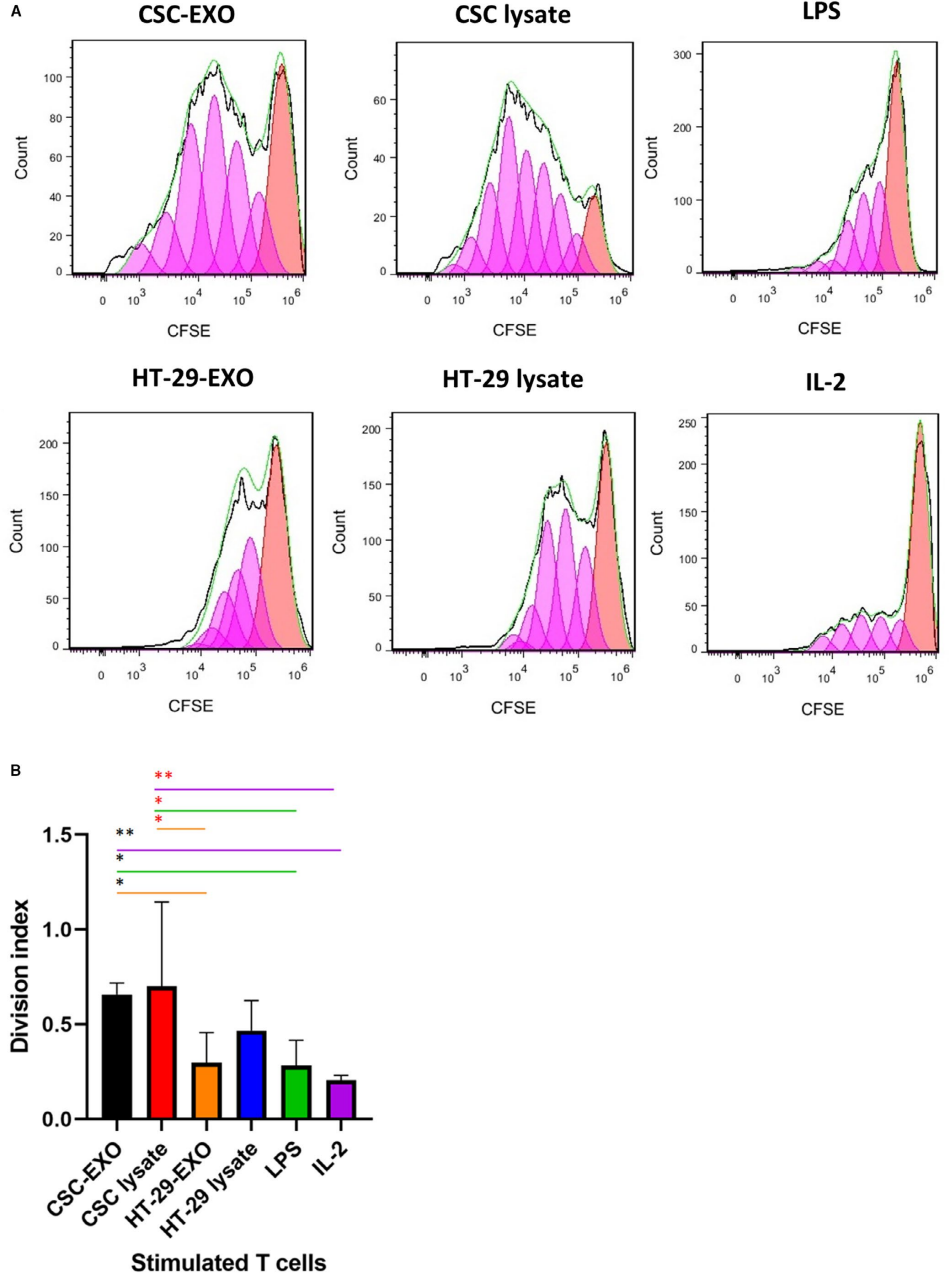
The proliferation of primed T cells in co‐culture with mature DCs. Human CFSE‐labelled T cells were primed using mature DCs from CSC_enr_‐EXOs and other antigen groups; following co‐culture with the same DCs for 5 d, the proliferation of CFSE‐labelled T cells was determined using flow cytometery. (A) Flow cytometery histograms of CFSE dilution from one representative experiment are shown. (B) Quantitative data from the division index (average division numbers that a cell in the original population had gone through; the average also takes into account the undivided cells) for each group from four independent experiments showing significance for the CSC_enr_‐lysate and CSC_enr_‐EXOs‐pulsed DCs. Data are presented as mean ± SD%

To examine the capacity of CSC_enr_‐EXOs‐treated DCs in induction of T‐cell proliferation and comparing it to the results of other groups, T cells were stimulated for 8 days by co‐culture with antigen‐loaded mDCs from the four antigen groups (Table 2). The primed T cells were labelled with CFSE and DC‐promoted T‐cell proliferation was evaluated by CFSE dilution of restimulated T cells via a second stimulation with accordingly loaded DCs for 5 days. IL‐2 treated T cells served as the positive control. As shown in Figure 4A,B, CSC_enr_‐EXO‐ and CSC_enr_ ‐lysate‐loaded DCs displayed a significantly higher proliferation rate than HT‐29‐EXOs, LPS‐alone and IL‐2‐treated (positive control) groups. On the contrary, the proliferation of T cells primed with HT‐29 lysate‐ or HT‐29‐EXO‐loaded DCs did not significantly differ from that of the LPS‐alone and the positive IL‐2 control groups. Thus, there was a most striking advantage of DC loaded with CSC_enr_ antigens in promoting T‐cell proliferation.

### 3.5 Cytotoxicity of CSC‐EXOs‐pulsed DCs activated T cells

To further monitor the functional consequence of DC‐based T‐cell priming in the context of different antigen groups (Table 2), we evaluated the cytotoxic activity of DC‐primed T cells towards CSC_enr_ spheres in the four antigen groups. CSC‐enriched spheroid targets were co‐cultured for 24 hours with restimulated T cells at a 1:10 (spheroid cells/primed T cells) ratio and the diameter of spheroids was investigated.

**FIGURE 5:**
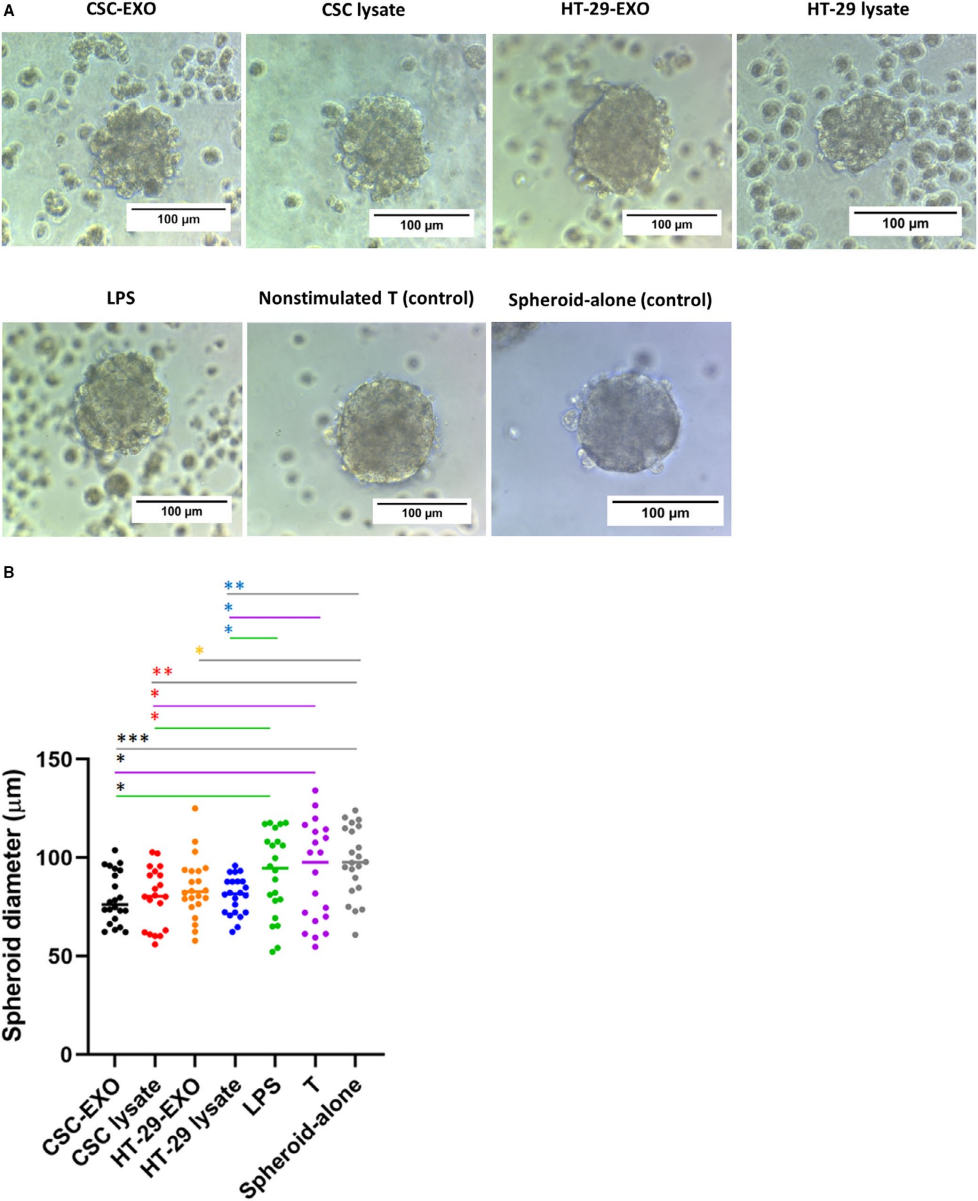
The effect of activated T cells on spheroid integrity and size. (A) Representative images from spheroids in different co‐culture groups. (B) Size comparison of spheroids indicated a significant decrease in spheroids diameter from CSC lysate, CSC‐EXOs and HT‐29 lysate antigen groups compared with the control groups. Data represent the mean diameter ± SD (n = 20)

Distinct to the co‐culture with IL‐2‐treated T cells, co‐culture with antigen‐stimulated T cells from all groups affected spheroid morphology with a reduction in size, disruption of the integrity of the spheroid surface and single cell dispersion (Figure 5A). This was confirmed by quantification of the spheroid size. All four antigen‐treated groups displayed smaller spheres compared to the control groups (spheroid‐alone, non‐stimulated T cells and LPS‐alone). Furthermore, cytotoxic activities of T cells stimulated by DCs from CSC_enr_‐EXOs slightly exceeded that of the remaining three groups and that of HT‐29‐EXO exhibited the weakest advantage compared to T cells stimulated in the absence of antigen. However, the latter disadvantage, which may be linked to the up‐regulation of IL‐10, was minor (Figure 5B). Thus, co‐culture with antigen‐loaded DC promotes cytotoxic T‐cell activation. Though differences between the four antigen groups did not reach a significant level, T cells primed with CSC_enr_‐EXO‐loaded DCs showed the strongest effect.

## 4 DISCUSSION

Great progress in immune response induction by tools as in vitro‐matured Ag‐pulsed DCs has opened promising paths for tumour immunotherapy, which was hampered for a long time by weak immunogenicity of most tumour‐associated antigens and immunosuppressive features of tumour cells.^45^, ^46^ However, tumours contain a large panel of tumour antigens, including patient‐ and even cancer type‐independent antigens that on the one hand may allow for expansion of multiple T‐cell clones giving whole tumour antigen‐based vaccines the capacity to foil the tumour cell escape, and on the other hand, may not be recovered in sufficient quantity to guarantee T‐cell activation (low zone tolerance induction). Particularly the latter obstacle is circumvented by the use of TEX,^44^, ^47^ these tiny vesicles being strongly enriched in tumour antigens compared to their parental cells. Vaccinations with TEXs have shown promising results in in vitro and pre‐clinical animal models, making TEXs potential candidates for triggering effective anti‐tumour immune responses.^31^, ^48^, ^49^ Cancer immunotherapy is confronted with a second handicap. Several studies, in various malignancies including colorectal cancer, demonstrated that the small population of CSCs are resistant to conventional therapies, contribute to tumour relapse and obviously escape whole tumour antigen vaccination.^9^, ^50^ This may by favoured by the instability of CSC with their capacity to differentiate and dedifferentiate, but more likely on CSC expressing antigens distinct from that of mature tumour cells,^51^ where it become likely that the amount of CSC antigens does not suffice for T‐cell activation due to the low number of CSCs. ^19^, ^21^, ^52^ However, to our knowledge, no study has thus far investigated the effect of utilizing CSC‐derived exosomes, as a novel Ag source, in induction of DC‐based anti‐tumour immune responses. One of the preliminary steps is the in vitro evaluation of the immunogenicity of this novel (tumour) Ag source in DC‐based priming of effector T‐cell responses,^53^ which we explored for CSC_enr_‐EXOs derived from human colon adenocarcinoma HT‐29 cell line with respect to their potential impact on DC maturation and subsequent T‐cell activation.

Tumour spheres are a well‐accepted, frequently used surrogate systems in the assessment of CSC‐related characteristics in in vitro and in vivo studies of cancer biology and treatment.^54^, ^55^ Accordingly, we generated spheroids from the HT‐29 cell line. With the exception of *NANOG*, spheroids showed significantly elevated levels of the *OCT4*, *KLF4* and *C‐MYC* key stemness genes and an increased frequency of CRC‐CSC markers, which reached significance for CD166 and CD133, supporting CSC enrichment in spheres.

Size distribution analysis of HT‐29‐EXOs and CSC‐EXOs isolated from the supernatant of HT‐29 spheroids corresponded to the features described for EXO and the constitutive EXO markers CD63 and CD81 were detected at an even higher level in CSC‐EXOs than HT‐29‐EXOs. Exosomes reflecting the state and specific content of the originating cell,^56^, ^57^, ^58^ the above outlined CSC marker enrichment appears promising towards CSC_enr_‐EXOs potentially providing CSC‐selective features that might promote DC maturation and activation and presentation of CSC‐specific antigens/peptides to CSC antigen‐specific T‐cell clones.

The DC maturation status plays a pivotal role in induction of immune responses following DC‐based vaccination,^59^, ^60^ where increased expression of key costimulatory and maturation molecules (*eg* CD86, CD40, HLA‐DR and CD83) involved in effector T‐cell activation is of major importance.^61^, ^62^ Our results regarding the impact of different antigen sources on DC phenotypic maturation status, as assessed by the expression level of CD40, CD86 and HLA‐DR, showed no significant differences compared to the positive control group (LPS‐alone). The CSC_enr_‐EXOs and also the additional Ag sources excluded undesired or suppressive impacts on the phenotypic maturation process of DCs. Cytokine delivery is another important feature of antigen‐loaded mature DC. DC can secrete IL‐10, which down‐regulates immune responses via exerting immunosuppressive impacts on both APCs and T cells.^63^, ^64^ In contrast, the secretion of pro‐immunogenic IL‐12 is critical for effective DC function and enhanced T‐cell stimulation.^65^, ^66^, ^67^ The four antigen sources did not differ significantly in IL‐12 secretion and stimulated IL‐12 secretion similar to LPS. Instead, IL‐10 secretion differed strikingly. Both, LPS and HT‐29‐EXOs promoted IL‐10 secretion far stronger than the remaining three antigen sources. Accordingly, though in all instances IL‐12 secretion exceeded IL‐10 secretion, the lowest IL‐12 to IL‐10 ratios were observed with LPS and HT‐29‐EXOs. A review article discussion on DC‐based therapies describes low‐level IL‐12 secretion by using exosomes for DC stimulation and suggests that exosomes might be a poorer antigen source than lysates.^67^ However, this has not been the case in our model. On the other hand, the high level of IL‐10 delivery by HT‐29‐EXO‐loaded DC might open a path towards immunosuppressive pathway stimulation by tumour cell exosomes, which could provide another explanation. As we did not observe high IL‐10 delivery by HT‐29‐derived CSC_enr_‐EXOs, a general disadvantage by DC stimulation via EXO can be excluded. Irrespective of this open question, it is important to note that DCs pulsed with CSC_enr_‐EXOs or CSC_enr_ lysates, but also of HT‐29‐EXOs or lysates favourably increase the IL‐12 to IL‐10 ratio compared to the LPS‐alone group. Thus, CSC_enr_‐EXOs are not only an efficient antigen source, but apparently are also poor carriers of immunosuppressive/immunosuppression stimulants, further supporting the potential capacity of CSC_enr_‐EXOs in the generation of potent mature DCs.

Having excluded a negative impact of CSC‐EXOs on DC activation, but gathering evidence for appropriate DC activation, the important question on T‐cell activation remained to be answered. We should stress, in advance, that we used restimulated T cells and that DC and T cells were derived from the same donor. We are aware that by the allogeneic origin of the 4 antigen sources some alloantigen‐specific T cells likely became activated. We are also aware that by the enrichment of CSC in spheres, we cannot differentiate between stimulation of tumour antigen‐ and CSC antigen‐specific T cells. Instead, despite the same status of allogeneic / HLA mismatching between T cells and spheroids in all antigenic‐ and control (T cells stimulated with IL‐2 alone or LPS‐matured DC) groups, there was a clear and strong ranking between antigen‐stimulated and control T cells, which implies selective expansion of HLA‐independent antigen‐specific clones. It is also worthwhile noting that the proliferation rate of CSC_enr_‐EXOs and lysates significantly exceeded that of HT‐29‐EXOs and HT‐29 lysates. This implies that CSC_enr_ clones contain besides bulk tumour‐derived antigens, CSC‐specific clones. One possible source could be embryonic markers that escaped negative thymic selection during T‐cell maturation.^68^ Furthermore, the slight increase in proliferation after stimulation with CSC lysates compared to CSC_enr_‐EXOs might rely on intracellular antigens or breakdown products derived thereof that are not recovered in exosomes. However, these differences were minor. Irrespective of the open question towards the specificity of the individual clones, the pronounced induction of T‐cell proliferation in response to CSC_enr_‐EXO‐loaded DC appears most promising for approaching concomitantly CSC and terminally differentiated tumour cell elimination by vaccination with CSC_enr_‐EXO‐loaded DC.

All targeting strategies including immunotherapy ultimately aim at effective tumour cell elimination, where CSC‐enriched spheroid models have been used repeatedly to investigate in vitro cytotoxicity.^43^, ^69^ We co‐cultured primed T cells with spheroids to assess spheroid destruction capacity via measuring spheroids diameter. The CSC_enr_‐EXOs, similar to CSC_enr_ lysate and HT‐29 lysate groups significantly affected spheroids. However, as outlined above, without peptide elution we cannot provide information on the number of clones and their peptide specificity. Nonetheless, we would argue that the excess of spheroid destruction by CSC_enr_‐EXOs compared to HT‐29‐EXOs indicates a contribution of ‘CSC‐specific’ CTL. We also cannot exclude a contribution of MHC peptide‐specific T cells. However, due to the low probability of alloreactivity (1%‐10%) in response to vaccines containing allogeneic MHC molecules,^70^, ^71^ the low expression of MHC molecules in CSCs which is insufficient for T‐cell priming,^72^, ^73^ and most importantly insignificant cytotoxicity in our IL‐2 and LPS control groups, direct T‐cell stimulation by the alloantigens is unlikely, which probably could rely on too small alloantigen amounts. Nonetheless, we want to mention a possible and advantageous contribution of allogeneic T cells. To circumvent tumour‐induced immunosuppression and insufficient T‐cell stimulation by DC vaccines, several groups explored vaccination with allogeneic tumour cell preparations as a HLA type‐independent vaccine for the generation of ‘tumour‐specific’ T cells in cancer immunotherapy.^74^, ^75^ In line to our observation, the results from animal models and clinical trials of allogeneic tumour cell line‐derived vaccines have indicated autologous DCs and T cells activation, where the anti‐tumour immune responses were not HLA‐restricted.^76^, ^77^, ^78^ The vaccine efficacy could be improved by providing additional immunostimulatory signals through alloreactive T cells secreting high levels of T‐cell activating cytokines, helping to induce allo‐ and auto‐antibodies, enhancing the cross‐priming of antigen‐presenting cells (APCs) for CD8+ T cells responding to tumour antigens.^78^, ^79^, ^80^, ^81^, ^82^, ^83^ The presence of allo‐MHC class II molecules may also be beneficial for Th activation.^84^, ^85^

Nonetheless, MHC antigens likely being lower in spheroid‐derived than in differentiated tumour cell exosomes and lysates, allogeneic cytotoxic T cells can be expected to be less prevalent after vaccination with CSC_enr_‐EXOs/lysates. Irrespective of this assumption, it should be pointed out that in the clinic CSC and exosomes derived thereof can be collected from the individual patient's peripheral blood concomitantly and from the same blood sample as the DC. Thus, without negotiating some advantages by an allogeneic vaccine, a fully autologous vaccination has the advantage of being tailored for the individual patient's tumour and CSC antigens. Nonetheless and by no means, we want to deviate from the necessity to define at least the major differentiated tumour cell‐ and CSC‐derived immunogenic peptides. First hints towards the mode and efficacy of CSC‐EXOs can be obtained by comparative proteomic analyses of CRC CSC‐EXO vs parental CRC‐EXO and non‐transformed colonic epithelial cell‐derived EXO. For a final proof, peptides of identified antigens need to be eluted from DC MHC‐I and MHC‐II molecules. Those studies also would unravel overlapping and distinct CSC and differentiated tumour cell antigens. Whether a repetition with CRC‐CSC or differentiated CRC cell lysates adds valuable information cannot, at present, be judged on and may for a vaccination approach not be necessary. Despite these missing informations, our data unequivocally demonstrate that vaccination with CSC_enr_‐ and HT‐29‐EXO‐loaded DC suffices for the activation of CRC‐specific CTL with a slight advantage of CSC_enr_‐EXO. Thus, CSC_enr_‐EXOs are a promising antigen sources to initiate a cytotoxic immune response against this subpopulation as well as terminally differentiated tumour cells.

Taken together, this first report on the immunogenic potential of sphere‐derived exosomes (CSC_enr_‐EXOs) unravelled that CSC‐EXOs support DC maturation and contain immunogenic antigens promoting anti‐tumour responses. In fact, CSC‐EXOs exert no suppressive/inhibitory effects on DC maturation, and even shift the IL‐12/IL‐10 ratio in favour of immunostimulation, accompanied by significant autologous T‐cell proliferation and spheroid‐directed cytotoxic T‐cell activation, the immune response induction efficacy of CSC‐EXOs being comparable or slightly superior to that of CSC_enr_ lysate. Furthermore, no disadvantages of CSC_enr_‐ compared to HT‐29‐EXOs/lysates were noted in DC maturation and T‐cell stimulation by DC. It remains to be proven, but appears most likely that the antigen profiles of CSC_enr_‐EXOs contain CSC‐ and differentiated cancer cell‐specific antigens. As far as this is not the case or the ratio is very imbalanced, a combination strategy making use of CSC_enr_‐ and HT‐29‐EXOs or CSC and HT‐29 lysates could be advantageous. We suggest that the explicit engagement of CSC in vaccination protocols provides a major breakthrough in cancer immunotherapy. With this in mind, CSC‐EXOs deserve extensive in vitro and in vivo investigations to unravel their content and their immunogenicity as a weapon against the tumour mass and the deleterious CSCs.

## CONFLICT OF INTEREST

The authors have no conflict of interest.

## AUTHOR CONTRIBUTIONS

**Marzieh Naseri:** Formal analysis (lead); investigation (lead); methodology (lead); software (equal); writing‐original draft (lead). **Margot Zöller:** Conceptualization (supporting); methodology (supporting); validation (supporting); writing‐review & editing (lead). **Jamshid Hadjati:** Conceptualization (supporting); methodology (supporting); writing‐review & editing (supporting). **Roya Ghods:** Conceptualization (supporting); methodology (supporting); validation (supporting). **Ehsan Ranaei Pirmardan:** Formal analysis (supporting); investigation (supporting); methodology (supporting); validation (supporting); writing‐review & editing (supporting). **Jafar Kiani:** Methodology (supporting); visualization (supporting). **Leila Eini:** Methodology (supporting); visualization (supporting). **Mahmood Bozorgmehr:** Conceptualization (lead); formal analysis (supporting); methodology (supporting); supervision (lead); validation (lead); writing‐review & editing (lead). **Zahra Madjd:** Conceptualization (lead); methodology (supporting); supervision (lead); validation (supporting); writing‐review & editing (lead).

## Data Availability

The data that support the findings of this study are available from the corresponding author upon reasonable request.
